# The effects of chronic AMPK activation on hepatic triglyceride accumulation and glycerol 3-phosphate acyltransferase activity with high fat feeding

**DOI:** 10.1186/1758-5996-5-29

**Published:** 2013-05-31

**Authors:** Bradley S Henriksen, Mary E Curtis, Natasha Fillmore, Brandon R Cardon, David M Thomson, Chad R Hancock

**Affiliations:** 1 Department of Nutrition, Dietetics, and Food Science, Brigham Young University, Provo, UT 84602, USA; 2 Department of Physiology and Developmental Biology, Brigham Young University, Provo, UT 84602, USA

**Keywords:** AMPK, GPAT1, SREBP-1c, mTOR, LCAD

## Abstract

**Background:**

High fat feeding increases hepatic fat accumulation and is associated with hepatic insulin resistance. AMP Activated Protein Kinase (AMPK) is thought to inhibit lipid synthesis by the acute inhibition of glycerol-3-phosphate acyltransferase (GPAT) activity and transcriptional regulation via sterol regulatory element binding protein-1c (SREBP-1c).

**Methods:**

The purpose of this study was to determine if chronic activation of AMPK prevented an increase in GPAT1 activity in rats fed a high fat diet. Rats were fed a control (C), or a high fat (HF) diet (60% fat) for 6 weeks and injected with saline or a daily aminoimidazole carboxamide ribnucleotide (AICAR) dose of 0.5 mg/g body weight.

**Results:**

Chronic AMPK activation by AICAR injections resulted in a significant reduction in hepatic triglyceride accumulation in both the C and HF fed animals (C, 5.5±0.7; C+AICAR, 2.7 ±0.3; HF, 21.8±3.3; and HF+AICAR, 8.0±1.8 mg/g liver). HF feeding caused an increase in total GPAT and GPAT1 activity, which was not affected by chronic AMPK activation (GPAT1 activity vs. C, C+AICAR, 92±19%; HF, 186±43%; HF+AICAR, 234±62%). Markers of oxidative capacity, including citrate synthase activity and cytochrome c abundance, were not affected by chronic AICAR treatment. Interestingly, HF feeding caused a significant increase in long chain acyl-CoA dehydrogenase or LCAD (up 66% from C), a marker of fatty acid oxidation capacity.

**Conclusions:**

These results suggest that chronic AMPK activation limits hepatic triglyceride accumulation independent of a reduction in total GPAT1 activity.

## Background

AMP-activated protein kinase (AMPK) is a major regulator of energy homeostasis and nutrient metabolism. AMPK is known to regulate fatty acid metabolism, protein synthesis, and glucose uptake [1,2]. Furthermore, the activation of AMPK occurs by allosteric and covalent modification of the enzyme in response to an energy deficit [3]. AMPK exerts its effects on energy metabolism by acutely regulating enzyme activity and protein abundance as well as influencing transcription and translation of genes involved in energy metabolism [4-6]. For these reasons, AMPK is of tremendous interest in understanding the mechanisms involved in hepatic lipid accumulation.

Hepatic lipid accumulation occurs in conditions of elevated dietary fat, obesity, and decreased metabolic function associated with decreased liver function. There are a number of mechanisms that could lead to increased hepatic lipid accumulation. Simply put, hepatic lipid accumulation is the result of a greater amount of lipid uptake and/or synthesis relative to lipid oxidation and release into the circulation [7,8]. Non-alcoholic fatty liver disease (NAFLD) is defined as hepatic fat accumulation greater than five percent of liver weight in the absence of excessive alcoholic intake [9]. Approximately 10-30 percent of the adult population in the United States is thought to have NAFLD, making it the most common chronic liver condition among adults [10-17]. It has also extended to adolescents with one study reporting approximately 61 percent of adolescent subjects with elevated liver enzymes (a marker of NAFLD) being overweight or obese, [18]. NAFLD is strongly associated with insulin resistance and is the hepatic representation of metabolic syndrome [9,11,19,20]. If not corrected, NAFLD can lead to the development of non-alcoholic steatohepatitis (NASH), cirrhosis of the liver and hepatocellular carcinoma [21].

Consistent with AMPK’s demonstrated role in energy metabolism, AMPK has been reported to increase lipid oxidation and inhibit lipid synthesis. One proposed mechanism for AMPK induced lipid regulation is in the acute inhibition of glycerol-3-phosphate acyltransferase (GPAT), an integral enzyme in triglyceride accumulation. GPAT is the rate-limiting enzyme catalyzing the first committed step in triglyceride synthesis [22,23]. Of the four predominant isoforms of GPAT, three are inhibited by *N*-Ethylmaleimide (NEM). In contrast, the isoform GPAT1 which is localized to the outer membrane of the mitochondria is resistant to NEM and accounts for 10 percent of the total GPAT activity in extra-hepatic tissues. In the liver, GPAT1 accounts for 30 to 50 percent of the total GPAT activity, making it a significant contributor to hepatic triglyceride regulation [24-26]. Chemical activation of AMPK by an AMP-analog aminoimidazole carboxamide ribonucleotide (AICAR) reduces fat accumulation in the hepatocyte by decreasing GPAT1 activity by 30 to 40 percent [5,23,26]. It is also likely that AMPK limits the fatty acid availability for triglyceride synthesis by increasing fat oxidation rates. AMPK inhibits acetyl-CoA carboxylase (ACC), an enzyme that catalyzes the formation of malonyl-CoA. Malonyl-CoA inhibits carnitine palmitoyltransferase I (CPT1) resulting in decreased beta-oxidation and increased fat synthesis. Decreasing malonyl-CoA production results in an increase in CPT1 activity [5,27-29]. Therefore, through AMPK’s acute role of inhibiting GPAT1 and increasing CPT1, there is an overall increase in oxidation relative to triglyceride synthesis.

In addition to acute regulation of triglyceride synthesis enzymes, recent evidence points to a role in which AMPK influences the transcription and translation of lipid synthesis enzymes [4,30]. Sterol regulatory element binding protein-1c (SREBP-1c) increases the transcription of lipid synthesis enzymes such as ACC, fatty acid synthase (FAS), GPAT, and stearoyl-CoA desaturase (SCD1) [13,31-34]. Previous work suggests that activation of AMPK decreases promoter activity of SREBP-1c in the liver cells, as well as decreasing the transcriptional activity of liver X receptors (LCRa), an upstream transcription factor and regulator of SREBP-1c expression, thus decreasing SREBP-1c and LXR [4]. Further, AMPK decreases SREBP-1c activity by interfering with the mammalian target of rapamycin complex (mTOR) activity. The proposed mechanism for mTOR-dependent activation of SREBP-1c is thought to be by cleavage of the SREBP-1c molecule [35]. Other studies done using cell culture models have shown that AMPK activation can inhibit mitochondrial GPAT1 abundance by decreasing SREBP-1c activity [4,36]. Thus, in addition to AMPK’s role as an acute regulator, AMPK may further inhibit hepatic lipid accumulation by inhibiting SREBP-1c through transcriptional regulation reduction of mTOR activity.

While there is some evidence for AMPK dependent inhibition of lipogenic enzymes [30,37], it is not completely understood how AMPK activation mediates this effect in liver tissue. Furthermore, a greater understanding of the role of AMPK activation in the process of hepatic lipid accumulation is becoming increasingly important due to the prevalence of NAFLD and NASH as noted above. AMPK may be a valuable therapeutic target for the treatment of these conditions. Therefore, the purpose for this study was to examine the effects of chronic activation of AMPK on enzymes critical for hepatic triglyceride accumulation and lipid synthesis, specifically ACC and GPAT1. This study was designed to gain a greater understanding of the role of chronic activation of AMPK on hepatic triglyceride synthesis and accumulation.

## Materials and methods

### Animal care

All procedures related to animal care and use were approved by the Institutional Animal Care and Use Committee of Brigham Young University.

### Diet

Male Wistar rats approximately 25 days old were divided into 4 groups of 7-10 animals each. Two groups consumed laboratory chow diet, 5053 PicoLab® Rodent Diet 20, and two groups consumed the high fat diet [38-40]. Food and water were provided *ad libitum*.

High Fat diet (g/kg of food):

116.3 g olive oil, 232.7 g flax seed oil, 87.2 g sugar, 174.6 g starch, 226.6 g casein, 4.5 g methionine, 30.7 g gelatin, 51.2 g bran, 22.5 g vitamin mix (Harlan Teklad, AIN76A), 52.2 g mineral mix (Harlan Teklad, AIN76), 1.4 g choline chloride. See Table 1 for the macronutrient composition of the two diets used in these studies.

**Table 1:** Macronutrient composition of diets

	**Chow diet (% of calories)**	**High fat diet (% of calories)**
Protein	23.6	20
Carbohydrate	64.5	20
Fat	12	60

### Experimental design

A two-factor research design was used to examine the effects of high fat feeding as well as AMPK activation on factors important for the determination of fat accumulation in liver tissue. See Table 2 for a complete description of control and treatment groups.

**Table 2:** Experimental design

**Chow groups**	**High fat groups**
Chow + Daily Saline Injections	High Fat (HF) + Daily Saline Injections
Chow + Daily AICAR Injections	High Fat (HF) + Daily AICAR Injections

### AICAR injections

AICAR was dissolved in 0.9% NaCl and administered subcutaneously every morning for six weeks at a dose of 0.5 mg AICAR/g body weight. Rats were sacrificed 24 hours after the last injection was given. Throughout the six weeks, rats from Control and High Fat groups were handled daily and injected with a comparable amount of saline at the time the AICAR groups received AICAR injections.

### Dissections

Rats were anesthetized with pentobarbital sodium (65 mg/kg body weight.) Liver tissue was extracted once rats were completely sedated. Liver was quickly removed and clamp frozen with liquid nitrogen chilled metal tongs then wrapped in aluminum foil and stored at -90 degrees Celsius.

### Citrate synthase activity

As a measure of mitochondrial oxidative capacity, whole tissue homogenate was used to measure citrate synthase activity by the method of Srere [41]. Briefly, homogenates were prepared in 175 mM KCl, 10 mM GSH, 2 mM Ethylenediaminetetraacetic acid (EDTA), pH 7.4 (100 mg of tissue per ml.) Homogenates were further diluted in a solution of 100 mM Tris pH 8.0. Citrate synthase activity was measured and recorded for each sample.

### Measurement of protein/enzyme abundance and phosphorylation

Standard bicinchoninic acid (BCA) protein assays were performed to determine protein concentration in the whole tissue or membrane fraction homogenate. The results were used to normalize protein content for Western Blots and activity assays.

Standard Western blotting procedures were performed. Briefly, aliquots of the liver homogenates were subjected to sodium dodecyl sulfate polyacrylamide gel electrophoresis (SDS-PAGE) and then transferred to a nitrocellulose membrane. The membrane was blocked with 5% milk solution followed by incubation with the primary antibody for the protein of choice overnight at 4 degrees Celsius. The next day, the membranes were rinsed with Tris-Buffered Saline Tween-20 (TBST) and incubated with the appropriate secondary antibody dissolved in 1% milk in TBST for 1 hour. This was followed again by rinses in TBST followed by one rinse in TBS. Protein content was detected by chemiluminescence. Protein band densitometry was quantified and analyzed with Image J and Alpha Ease software [4].

Primary antibodies for the following proteins were used: Total AMPK (Cell Signaling; cat. No. 2532 L), phospho-AMPK (Cell Signaling, Berveraly, MA; cat. No. 2535 L), total ACC (GE Healthcare; cat. No. RPN1231V), SREBP-1c (Santa Cruz, Santa Cruz, CA; cat. No. SC13551), mTOR (Cell Signaling Technology Beverly, MA; cat. No. 2972 ).

In addition, we looked at 4E-binding protein (4E-BP) (Cell Signaling Technology Beverly, MA; cat no. 9452) and phosphor-raptor (Cell Signaling Technology Beverly, MA; cat no. 2083) for indications of mTOR activity and Cytochrome C (Santa Cruz, Santa Cruz, CA; cat. No. sc-13156) and LCAD (a gift from Daniel P. Kelly) for an indication of oxidative capacity.

### Triglyceride assay

Liver triglycerides were measured according to the Folch method. Forty to fifty milligrams of frozen liver were homogenized with a 2:1 concentration of chloroform:methanol and agitated in a cold room overnight at 4 degrees C. One ml of 0.9% NaCl was added to each sample solution, vortexed and centrifuged for 1 hour at 1000 × g at 4 degrees C. The organic phase was removed and dried down in a lyophilizer. Samples were reconstituted with 100 μl of Tert-Butanol Triton X solution. Trigylceride content was then analyzed in each sample with the WAKO triglyceride assay kit as per manufacturer’s instructions [42].

### GPAT activity assay

The activity of microsomal and mitochondrial GPAT was measured using the method described previously [5,43,44]. Briefly, membrane fractions of liver tissue were formed using sample homogenates prepared with 25 mg of liver and homogenization buffer (250 mM Sucrose, 10 mMTrisHCl, 1 mM dithiothreitol (DTT), 1 mM EDTA, pH 7.4). Homogenate was centrifuged at 100,000 x g for 1 hour, after which the supernatant was removed and the pellet reconstituted with 400 μl of the same homogenization buffer, aliquoted and frozen in -90 degree Celsius freezer. Total GPAT activity was measured using a cocktail of 800 μM [^14^C]glycerol 3-phosphate (G-3-P), 60 μM palmitoyl Co-A, 75 mM TrisHCl, 4 mM MgCl, 2 mg/ml BSA, 8 mM NaF, 1 Mm DTT, and non-labeled G3P. Sample (40 μg protein) was added to the solution incubated with and without 2 mM N-ethylmaleimide (NEM), an inhibitor of microsomal GPAT to isolate and measure the remaining activity, GPAT1 activity. The reaction was run for 10 min at 37°C and stopped with 0.6 ml 1% HClO_4_ and chloroform-methanol (2:1). After 5 minutes on ice, another 1 ml of 1% perchloric acid and 1 ml of chloroform was added to the solution. Samples were centrifuged for 1 hour at 1000 × g and washed 3 times with 1% perchloric acid after which 1 ml of organic phase containing the labeled G-3-P incorporated into lysophosphatidic acid was dried down using a lyophilizer. After reconstituting the samples with 2:1 tert-butanol triton × solution, the sample with scintillation fluid was placed into scintillation tubes and counts measured with a scintillation counter. Subtracting the mitochondrial GPAT (NEM-resistant) activity from the total activity allowed for determining GPAT1’s activity for each sample.

### Statistical analysis

Significant differences between groups were determined using two way analysis of variance (ANOVA) and Bonferonni post hoc test for multiple comparisons. The statistical software SigmaStat (Systat Software Inc, San Jose, CA) was used. Statistical significance is defined at p<0.05. Results are presented as means ± standard error of mean (SEM).

## Results

### Chronic activation of AMPK limits hepatic triglyceride accumulation

**Figure 1:**
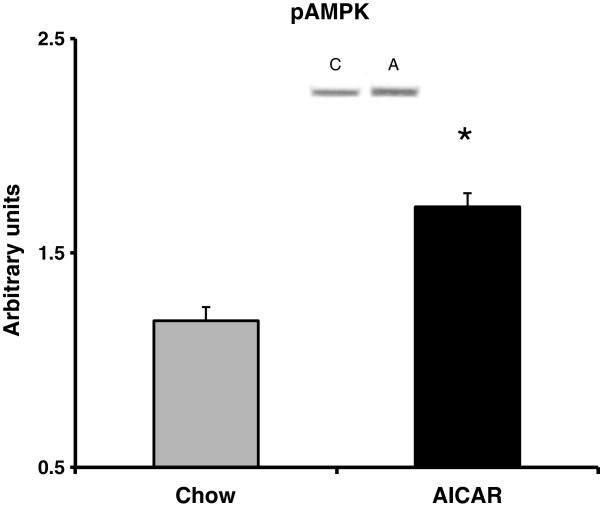
**Phospho-AMP-activated protein kinase (pAMPK) content in the liver was increased with acute 5-aminoimidazole-4-carboxamide riboside (AICAR) treatment (n=5-7).** Livers from AICAR-treated rats were removed 1 hr after injection. Asterisk (*) denotes a main effect of AICAR (p<0.05). Graph represents means ± SE.

**Figure 2:**
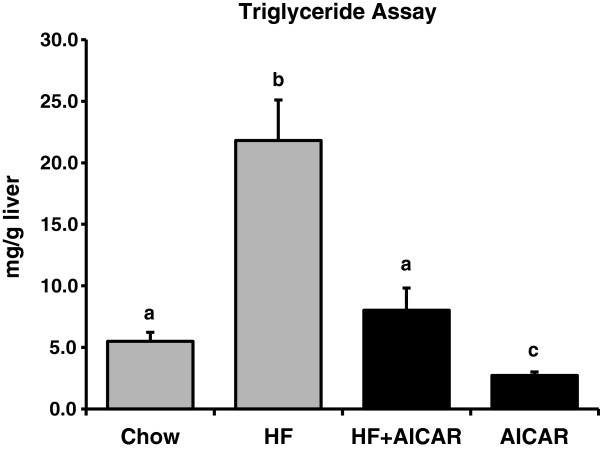
**Chronic activation of liver AMPK with daily injections of AICAR limited the normal increase in triglyceride accumulation that occurs with high fat feeding such that it was not significantly different from the control group (n = 7-12).** Letters are used to represent significance; same letter means no significant difference (P < 0.05). Graph represents means ± SE.

We have previously reported that rats given the same treatment as in this study exhibit a significant main effect of high fat feeding on increased circulating FFA’s and abdominal fat accumulation [39]. In addition, this duration of feeding as well as the dose and frequency of AICAR treatment does not result in a significant increase in body weight due to high fat feeding compared to control fed rats [39]. However, as we expected, chronic activation of AMPK using daily AICAR injections caused a reduction in hepatic triglyceride content. We verified that the subcutaneous AICAR injections at the dose we used (0.5 mg/g body weight) were sufficient to cause activation of AMPK in the liver by measuring AMPK phosphorylation one hour after acute subcutaneous injection in rats (see Figure 1). Chronic activation of AMPK limited the normal increase in triglyceride accumulation that occurs with high fat feeding such that it was not significantly different from the control group (See Figure 2). This finding is consistent with previous reports on the effects of AMPK activation on fat accumulation in the liver [45-47]. The mechanism by which AMPK causes this reduction in a prolonged treatment of six weeks has yet to be fully characterized.

### Regulation of lipid synthesis

#### Chronic activation of AMPK decreased SREBP-1c in livers of rats fed a high fat diet

**Figure 3:**
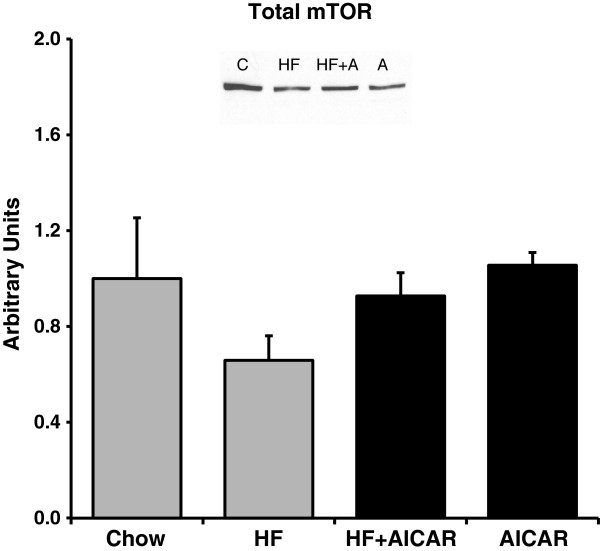
**Total mammalian target of rapamycin complex (mTOR) protein content of liver extracts revealed no significant differences with high fat feeding or chronic AMPK activation (n = 4-5).** Bands for all 4 groups were taken side by side with no interruption. Graph represents means ± SE.

**Figure 4:**
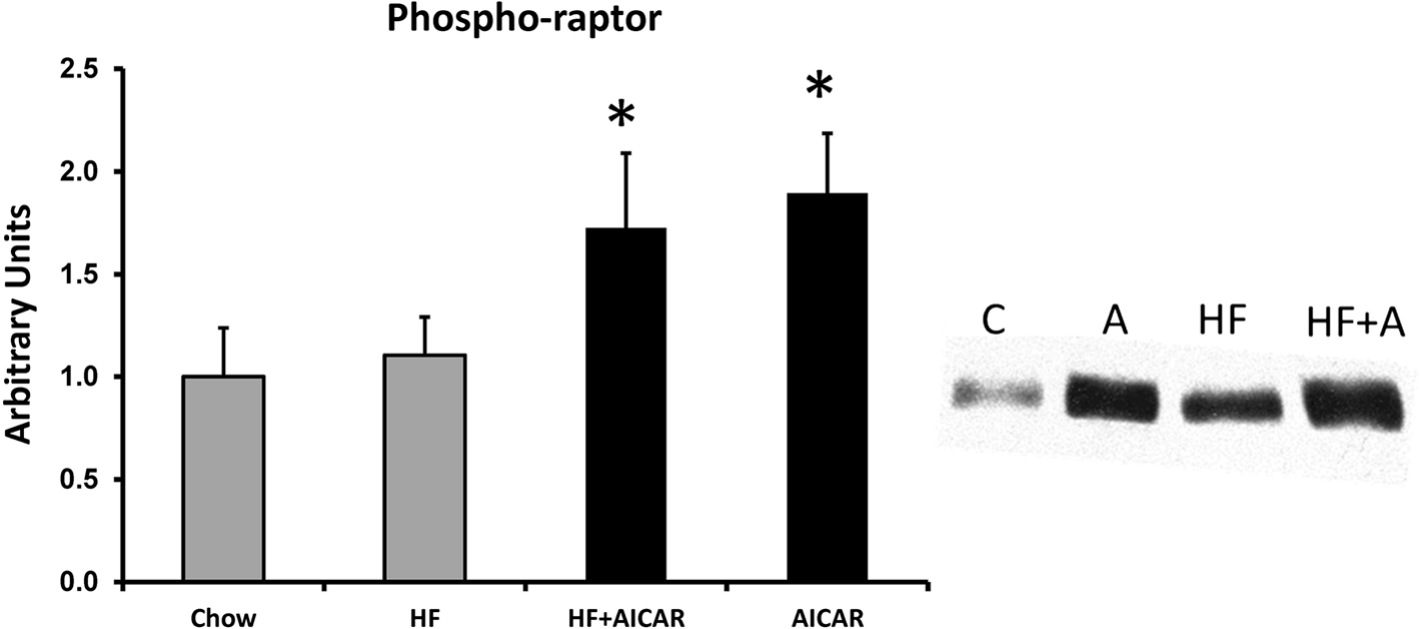
**Phospho-raptor content was increased in livers treated with AICAR (n= 4-5).** Asterisk (*) denotes a main effect of AICAR (p<0.05). Graph represents means ± SE.

**Figure 5:**
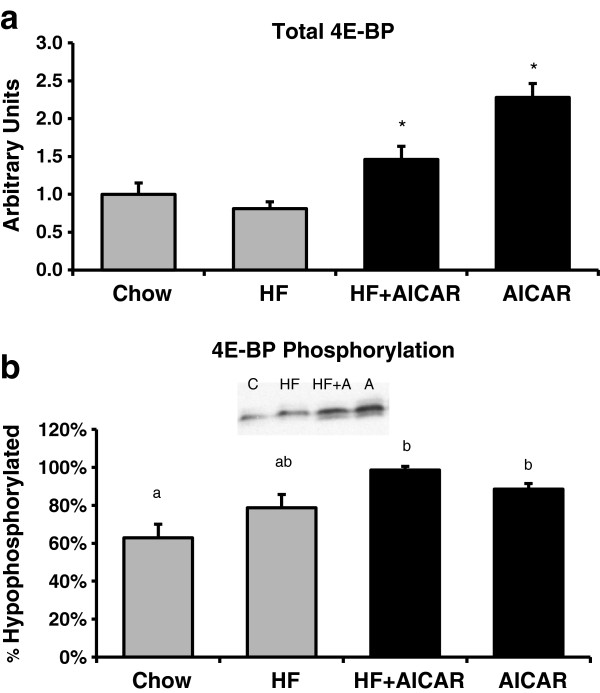
**Eukaryotic initiation factor 4E-binding protein (4EBP) following HF and AICAR treatments.a.** Total Eukaryotic initiation factor 4E-binding protein (4EBP) results show an increase with the treatment of AICAR (n = 4-5). Bands for all 4 groups were taken side by side with no interruption Asterisk (*) denotes a main effect of AICAR treatment (P<0.05). Graph represents means ± SE. **b** Eukaryotic initiation factor 4E-binding protein (4EBP) phosphorylation (percentage of the total protein in the 2 hypophosphorylated bands compared to total) showed an increased phosphorylation with AICAR treatment (n = 4-5). Bands for all 4 groups were taken side by side with no interruption. Letters are used to represent significance; same letter means no significant difference (P < 0.05). Graph represents means ± SE.

Based on previous findings, chronic AMPK activation would be expected to reduce transcription of GPAT via inhibition of mTOR and SREBP-1c [4,35]. AMPK is known to inhibit mTOR activity therefore we examined mTOR, an mTOR regulatory protein (raptor), and a downstream target of mTOR (4EBP) as an indication of mTOR activity [48]. Western blots on total mTOR complex protein in each group did not indicate a significant difference between the groups (See Figure 3). As would be anticipated from the activation of AMPK, the total levels of phospho-raptor were significantly increased with chronic AMPK activation with both chow and high fat feeding (See Figure 4). Phosphorylation of raptor on (S792) by AMPK is important for mTOR inhibition [49-51]. The total abundance of 4E-BP was significantly increased with chronic AMPK activation with both the chow and high fat feeding (See Figure 5a.) The phosphorylation state was determined by the shift in molecular weight of total 4E-BP protein (percentage of the total protein in the two hypophosphorylated bands migrated down 1-2 kDa compared to the total at molecular weight of 20 kDa as previously documented) [48,52]. Considering the shift of 4EBP, our results indicate that there a was lower amount of phosphorylated 4EBP following chronic AICAR treatment compared to the control group, which is consistent with the known inhibitory effect of AMPK on mTOR activity (See Figure 5b).

**Figure 6:**
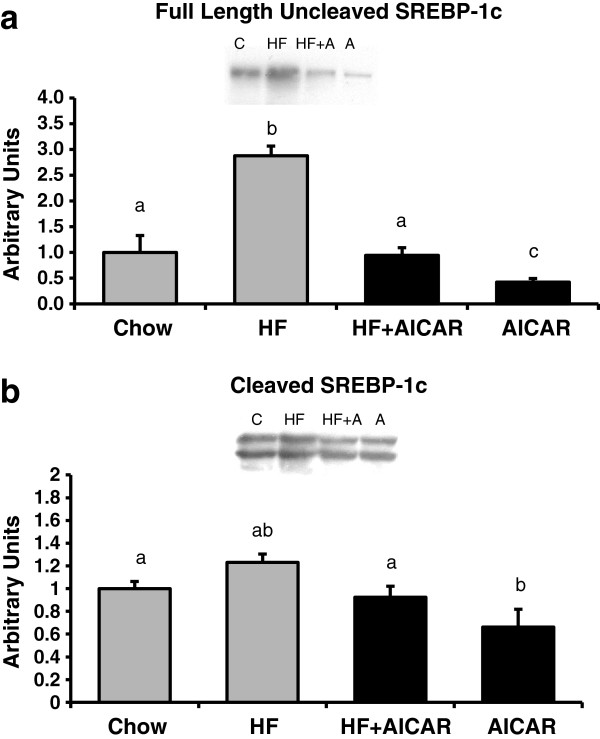
**Chronic activation of AMPK and sterol regulatory element binding protein-1c (SREBP-1c). a.** Chronic activation of AMPK caused a reduction in the total abundance of uncleaved Sterol regulatory element binding protein-1c (SREBP-1c) in rats fed either chow or high fat diet (n = 3-5). Bands for all 4 groups were taken side by side with no interruption. Letters are used to represent significance. A significant main effect of AICAR was observed (p<0.05). Graph represents means ± SE. **b**. Chronic activation of AMPK caused a reduction in the total abundance of cleaved (65-68 kDa bands) SREBP-1c in the liver of rats fed either chow or high fat diet (n = 4-5). Bands for all 4 groups were taken side by side with no interruption. Letters are used to represent significance. A significant main effect of AICAR and high fat feeding was observed (p<0.05). Graph represents means ± SE.

AMPK plays a major role in the activity of SREBP-1c in the liver by inhibiting mTOR complex activity [37]. SREBP-1c is positively regulated by mTOR and therefore lipogenesis is upregulated with increased mTOR activity [35]. We examined both forms of SREBP-1c in our study: the full-length (inactive) form, and cleaved (active) form [53]. High fat feeding caused a marked increase in total full-length, uncleaved SREBP-1c abundance. Consistent with the pattern observed in hepatic triglyceride accumulation, chronic activation of AMPK caused a reduction in the total full length, uncleaved SREBP-1c abundance in rats fed either chow or high fat diet (see Figure 6a). The cleaved SREBP-1c showed increases with high fat feeding and decreases with chronic AMPK activation as well but the differences were not as pronounced (See Figure 6b). Therefore, our data indicates that chronic activation of AMPK inhibits both full length and cleaved SREBP-1c protein abundance; this was consistent with what we observed with the mTOR dependent response as seen with 4EBP phosphorylation.

#### Chronic activation of AMPK had no effect on GPAT1 activity but a high fat feeding effect was present

**Figure 7:**
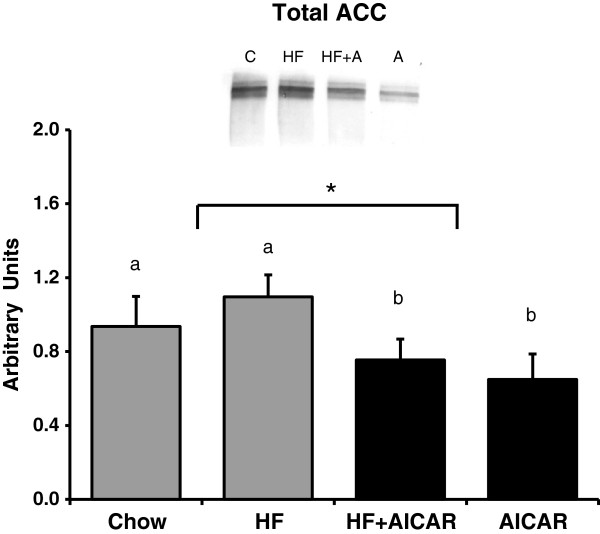
**Total acetyl coA carboxylase (ACC) content had a main effect of chronic AMPK activation (n = 7-10).** High fat feeding blunted the decrease in total ACC content with the HF + AICAR group. Bands for all 4 groups were taken side by side with no interruption. Letters are used to represent significance. Asterisk (*) denotes a main effect of AICAR (P < 0.05). Graph represents means ± SE.

Lipid synthesis enzymes increased by SREBP-1c include ACC and GPAT. We first examined the abundance of total ACC in response to high fat feeding and chronic AMPK activation and found that AMPK activation caused a significant reduction in total ACC protein in the chow group. Interestingly, high fat feeding did not produce a significant increase in total ACC protein (See Figure 7). These results are consistent with cleaved SREBP1-c total content.

**Figure 8:**
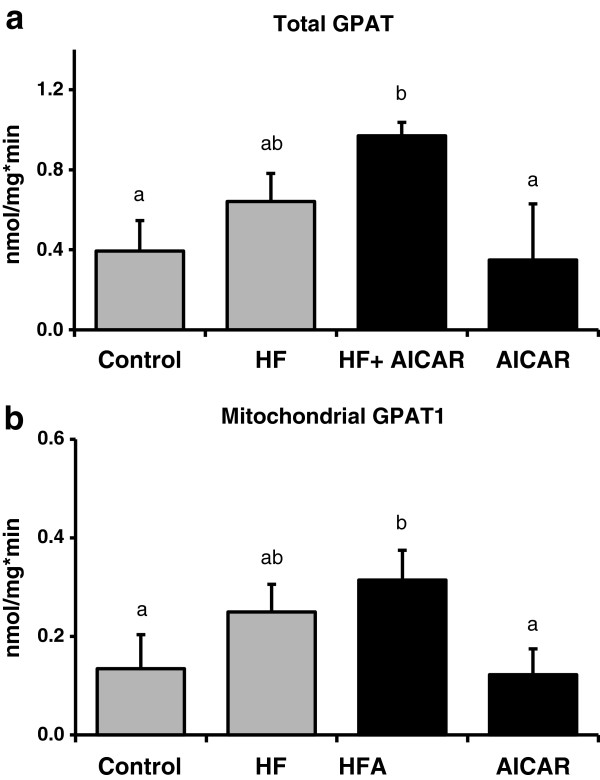
**GPAT activity following high fat feeding and AICAR treatments.a.** High fat feeding increased total glycerol-3-phosphate acyl-transferase (GPAT) activity (n = 5-8) in liver. A main AICAR effect on total GPAT activity was absent. Asterisk (*) denotes a main effect of high fat feeding (P < 0.05). Graph represents means ± SE. **b.** High fat feeding increased NEM-sensitive glycerol-3-phosphate acyl-transferase (GPAT1) activity in liver (n = 5-8). A main AICAR effect on total GPAT activity was absent. *Main treatment effect0020 (P < 0.05). Graph represents means ± SE.

GPAT1 activity was measured because it is another lipogenic target of SREBP-1C and is a rate-limiting enzyme for triglyceride synthesis [36]. High fat feeding caused an increase in total and NEM-sensitive GPAT activity in the liver (Figure 8a). Surprisingly, chronic activation of AMPK in either control or high fat fed animals did not cause a reduction in total or NEM-sensitive (GPAT1) activity (Figure 8b). Our results present the novel finding that there is not a direct correlation of chronic activation of AMPK with GPAT1 activity. We expected to see a reduction in GPAT1 activity based on previous results in hepatocytes regarding the acute effect of AMPK on GPAT activity [5,26]. These findings prompted further exploration of the mechanisms and regulation of fatty acid oxidation.

### Lipid oxidation

#### Long chain acyl-CoA dehydrogenase (LCAD) was not influenced by chronic AMPK activation but was increased with high fat feeding

**Figure 9:**
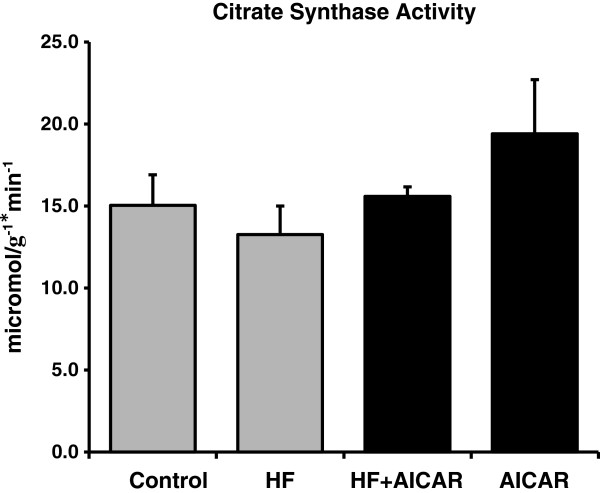
**Citrate synthase activity in the liver did not increase with either high fat feeding or chronic AMPK activation (n = 4-5).** Graph represents means ± SE.

**Figure 10:**
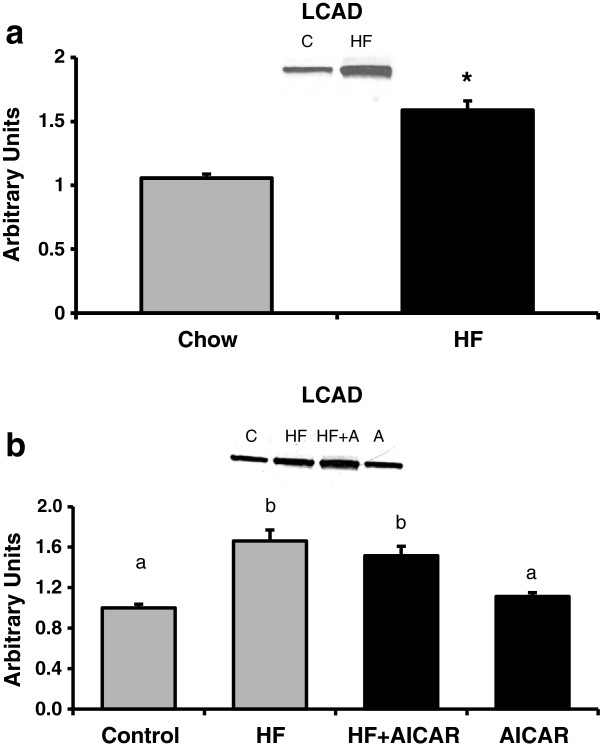
**Long chain acyl CoA dehydrogenase (LCAD) following high fat feeding and AICAR treatments. a.** There was a main high fat effect on total long chain acyl CoA dehydrogenase (LCAD) content in the liver (n = 8-10). Asterisk (*) indicates a main effect of high fat diet (P < 0.05). Graph represents means ± SE. **b.** Chronic activation of AMPK did not have an effect on total long chain acyl CoA dehydrogenase (LCAD) content in the liver (n = 4-5). Bands for all 4 groups were taken side by side with no interruption. Letters are used to represent significance; same letter means no significant difference (P < 0.05). Graph represents means ± SE.

Hepatic lipid accumulation is a balance between the lipid synthesis and oxidation so two markers of mitochondrial oxidative capacity in the liver were measured. Neither high fat feeding nor chronic activation of AMPK showed statistically significant differences between groups for citrate synthase activity (See Figure 9) or cytochrome c content (data not shown) in the liver. Long chain acyl-CoA dehydrogenase (LCAD), a key enzyme responsible for the first step in the oxidation of long-chain fatty acyl-CoAs was measured [54]. A significant increase in LCAD was observed in response to high fat feeding, suggesting greater capacity for fat oxidation (See Figure 10a). Interestingly no effect was seen in the animals treated with AICAR (See Figure 10b). These results suggest that chronic AMPK activation does not play a significant role in changing the oxidative capacity of liver content contrary to what has been found previously in skeletal muscle [38,39,55]. Similar to the GPAT1 data, these results do not account for the observed difference in hepatic triglyceride levels in response to chronic AMPK activation.

## Discussion

The purpose of this study was to examine a potential mechanism by which chronic AMPK activation limits fat accumulation in the liver. We hypothesized that AMPK would cause a reduction in GPAT1 activity, a rate limiting enzyme for triglyceride synthesis. GPAT1 has been shown to be influential in the development of NAFLD through its role in triglyceride synthesis in the liver and when overexpressed leads to excess triglyceride synthesis [22,26,56-58]. Neschen et al. and Hammond et al. demonstrated that GPAT1 knockout mice had less triglyceride accumulation when compared to wild type mice fed a high fat diet [59,60]. AMPK is thought to inhibit the transcription of GPAT by reducing the activation of SREBP-1c. SREBP-1c is the primary transcription factor for GPAT1 and other lipogenic enzymes [31-33]. In contrast to our hypothesis, we found that chronic AMPK activation did not cause a reduction in GPAT1 activity in either the control group or the animals receiving a high fat diet. Therefore, results from this study suggest that chronic AMPK activation limits triglyceride accumulation in the liver by a mechanism other than a reduction in triglyceride synthesis capacity.

It is well documented that AMPK activation reduces hepatic triglyceride accumulation [46,47]. However the mechanisms responsible for this reduced triglyceride content in the setting of high fat feeding are not fully understood. AMPK has been best characterized as a regulator of fatty acid oxidation [61]. AMPK affects an increase in oxidation by inhibition of ACC [47]. Inhibition of ACC results in less malonyl-CoA synthesis leading to a greater activity of CPT1 due to reduced inhibition by malonyl-CoA [28,61,62]. Recently, a greater appreciation of AMPK as a regulator of triglyceride synthesis has developed. Sterol regulatory element binding protein-1c (SREBP-1c) is a major regulator of lipogenic enzymes and AMPK reduces SREBP-1c and downstream lipogenic enzymes through an mTOR-dependent mechanism [4,47]. GPAT1 is one lipogenic enzyme that has been clearly associated with an increase in triglyceride synthesis and accumulation. The regulation of GPAT1 by SREBP-1c is evidenced by the 6.7-fold increase in GPAT1 by an overexpression of SREBP-1c in adipocytes [36]. Further, an expected increase in GPAT1 with re-feeding does not occur in liver in the absence of SREBP-1c [63]. Results from our study confirm that AMPK activation leads to a reduction in SREBP-1c abundance. Consistent with this reduction in SREBP-1c content, we observed an AMPK dependent reduction in total ACC, one of the enzymes positively regulated by SREBP-1c. GPAT1 activity assay results were unexpected and differed from the pattern observed with triglycerides, SREBP-1c and ACC. Total and NEM-sensitive (GPAT1) GPAT activity was increased with high fat feeding but chronic AMPK activation did not appear to have an inhibitory effect. Therefore our results in intact liver with chronic AMPK activation suggest an alternative regulation of total triglyceride synthesis capacity via GPAT1 than has previously been proposed in isolated hepatocytes with acute AMPK activation.

The regulation of SREBP-1c by AMPK is thought to be dependent upon inhibition of mammalian target of rapamycin (mTOR) and transcriptional activity of liver X receptor (LXR) and SREBP-1c [4,35,64]. SREBP-1c is significantly decreased by inhibitors of mTOR such as rapamycin. [65]. This indicates that through AMPK’s inhibition of mTOR activity, AMPK has the effect of reducing SREBP-1c activity Further, AMPK’s role in reducing mTOR activity results in decreased protein synthesis in liver tissue [48]. The mechanism by which AMPK decreases mTOR activity was proposed by Inoki et al. to be by phosphorylation and activation of an upstream protein in the signaling cascade, tuberous sclerosis complex 1/2 (TSC1/2) [66,67]. mTOR phosphorylates downstream proteins such as eukaryotic translation initiation factor 4E-binding protein (4E-BP) and ribosomal protein p70 S6 (S6K1) thereby increasing translation of various proteins and overall protein synthesis [48,68]. Therefore, we can get an indication of the effect of chronic AMPK activation on mTOR activity by measuring the phosphorylation state (or shift) of 4E-BP [48,52]. Our study validated the effect of AMPK activation in the liver by showing a decrease in phosphorylated 4E-BP (increased hypophosphorylation) in the AICAR treated groups [52]. This suggests an inhibition of mTOR activity and explanation for the pattern seen in the SREBP-1c results.

Triglycerides accumulate in the liver particularly with chronic high fat feeding through an up-regulation of lipogenic enzymes that enhance fatty acid and triglyceride synthesis and greater inhibition of CPT-1, a major regulator of beta-oxidation. This is evidenced by a marked decrease in beta oxidation when GPAT1 is overexpressed in hepatocytes [56,58,69] and increased beta oxidation markers when GPAT1 is knocked out in mouse myocytes [59]. There is clear evidence that a chronic high fat diet results in significantly higher hepatic weights and triglyceride levels [8,70,71]. Our study duplicated such results with an increase in triglycerides after prolonged high fat feeding. In accordance with reported results of AMPK activation in cultured hepatocyte models [45,72], the chronic AICAR treated intact liver tissue in our study had reduced levels of triglycerides in the liver to control levels. AMPK activation inhibits triglyceride accumulation by increasing beta oxidation in the cell [47,73] as well as in its proposed inhibition of mTOR and downstream targets such as SREBP-1c as noted above [67,74]. These mechanisms could explain the fat accumulation with high fat feeding and reductions with chronic AICAR treatment in the livers that was seen in our triglyceride assay results. Therefore, the reduction seen in triglyceride accumulation with chronic AMPK activation was consistent with what was expected.

Increased fat oxidation with high fat feeding could be another contributing factor to explain the conflicting findings of triglyceride content and GPAT1 data in our study. High fat states, such as ob/ob models, have shown an increase oxidative capacity with a simultaneous increase in fatty acid oxidation [75]. This high fat effect on fatty acid oxidative capacity gave reason for measuring LCAD, a marker of fatty acid oxidative capacity. Further, AMPK activation is known to influence mitochondrial biogenesis in both skeletal muscle [38,39,55] and in adipose tissue [76]. Interestingly, we did not see an increase in either citrate synthase activity or cytochrome c content with either high fat feeding or chronic activation of AMPK in the liver. However, a significant increase of LCAD with high fat feeding was observed. The increase seen in LCAD is consistent with the high fat effect expected but the chronic AMPK activation effect was not apparent from the data. Therefore, the issue of how the chronic effects of AMPK activation lead to a decrease in hepatic triglyceride accumulation remains to be resolved.

It is important to note some of the limitations in our study. First, our study did not investigate the acute regulation of GPAT by AMPK noted in other studies. AMPK has been shown to have an acute inhibitory effect on GPAT1 activity as shown in previous studies, which is likely due to phosphorylation of GPAT1 [5,24,26,77]. This acute effect was not the focus of our study, and it is not known whether this played a factor in overall triglyceride accumulation. The reduction in triglycerides could be explained solely by the acute inhibition of GPAT by AMPK. Second, the fat in the high fat diet used in this study was composed of olive oil and flaxseed oil and was not a typical composition for a high diet (see Methods) due to use of tissues from animals in a companion study. This may influence fat accumulation patterns seen in our study and/or responsiveness to AMPK. Therefore more work could be done to see if our results in the chronic setting were unique to the type of fat used in our study.

## Conclusions

Given the current trends in lifestyle and dietary habits, the prevalence of NAFLD and the development of NASH are likely to continue to increase. AMPK is widely recognized as a central regulator/sensor of cellular energy metabolism and when activated, is known to limit hepatic fat accumulation. Thus, AMPK is an attractive target for potential therapeutic interventions designed to treat excess fat accumulation in the liver.

In this study we examined the effect of chronic activation of AMPK via systemic administration of AICAR on hepatic GPAT1 activity. High fat feeding resulted in increased hepatic GPAT1 activity while chronic AICAR administration had no effect on GPAT1 activity over time. This was in contrast to the clear effect of AICAR treatment on triglyceride content and transcriptional regulation in the liver. Further areas of interest that may help explain our findings include research on the role of AMPK activation and other lipid regulatory mechanisms such as cellular uptake or release of fatty acids and other lipid molecules.

## Abbreviations

4E-BP: 4E-binding protein; ACC: Acetyl CoA carboxylase; AICAR: Aminoimidazole carboxamide ribonucleotide; AMPK: AMP Activated Protein Kinase; BCA: Bicinchoninic acid; C: Control; CPT1: Carnitine palmitoyltransferase 1; DTT: Dithiothreitol; EDTA: Ethylenediaminetetraacetic acid; FAS: Fatty Acid Synthase; G-3-P: [^14^C]glycerol 3-phosphate; GPAT: Glycerol-3-phosphate acyltransferase; GPAT1: N-ethylmaleimide resistant glycerol-3-phosphate acyltransferase; HF: High Fat; LCAD: Long chain acyl-CoA dehydrogenase; LCRa: Liver x receptors; LXR: Liver x receptor; mTOR: Mammalian target of rapamycin; NAFLD: Non-alcholic fatty liver disease; NEM: N-ethylmaleimide; S6K1: Ribosomal protein p70 S6; SCD1: Stearoyl-CoA desaturase; SDS-PAGE: Sodium dodecyl sulfate polyacrylamide gel electrophoresis; SREBP-1c: Sterol regulatory element binding protein; TBS: Tris-Buffered Saline; TBST: Tris-Buffered Saline Tween-20; TSC1/2: Tuberous sclerosis complex 1/2.

## Competing interests

Bradley Henriksen, Mary Curtis, Natasha Fillmore, Brandon Cardon, David Thomson, Chad Hancock. The authors declare that they have no competing interests.
